# Application of Composite Small Calibration Objects in Traffic Accident Scene Photogrammetry

**DOI:** 10.1371/journal.pone.0127018

**Published:** 2015-05-26

**Authors:** Qiang Chen, Hongguo Xu, Lidong Tan

**Affiliations:** School of Transportation, Jilin University, Changchun, Jilin, China; Politehnica University of Bucharest, ROMANIA

## Abstract

In order to address the difficulty of arranging large calibration objects and the low measurement accuracy of small calibration objects in traffic accident scene photogrammetry, a photogrammetric method based on a composite of small calibration objects is proposed. Several small calibration objects are placed around the traffic accident scene, and the coordinate system of the composite calibration object is given based on one of them. By maintaining the relative position and coplanar relationship of the small calibration objects, the local coordinate system of each small calibration object is transformed into the coordinate system of the composite calibration object. The two-dimensional direct linear transformation method is improved based on minimizing the reprojection error of the calibration points of all objects. A rectified image is obtained using the nonlinear optimization method. The increased accuracy of traffic accident scene photogrammetry using a composite small calibration object is demonstrated through the analysis of field experiments and case studies.

## Introduction

Traffic accident scene photogrammetry typically involves using a non-metric camera to capture accident scene photographs, and computer processing to measure important accident scene information [1–5]. Non-metric camera calibration is an important part of photogrammetry that directly affects the accuracy of the measurements. Depending on the availability of a high-precision calibration objects of known structure, there are three possible methods of calibrating a non-metric camera: traditional calibration [6, 7], self-calibration [8, 9], and active vision calibration [10–12]. Traditional calibration involves placing a calibration object in front of the camera, with a number of calibration points whose accurate 3D coordinates are known. The camera’s parameters are calculated by establishing the relationship of the coordinates of each point in space with their corresponding image coordinates. Self-calibration does not require the 3D coordinates of the calibration points, but it does need multiple images with corresponding coordinate points to determine the camera’s parameters. Active vision calibration requires the camera to move along a certain trajectory, and uses the geometric characteristics of the trajectory and relations between image coordinates to solve the camera’s parameters. The traditional method using calibration objects is generally used for traffic accident scene photogrammetry [13–15]. The two-dimensional direct linear transformation (2D-DLT) method based on a plane calibration object is simple, practical, and highly accurate. Thus, it is widely used at present [16, 17]. However, the 2D-DLT algorithm can also correctly reflect the perspective projection relationship between a spatial plane and the image plane of an entire scene, which requires as many calibration objects as possible to cover the site area of interest [18, 19]. A traffic accident scene can range up to dozens of meters, and difficulties exist in manufacturing and maintaining large high-precision calibration objects to match the site. However, a small calibration object can only offer a small range of feature point data, which leads to a reduction in the photographic measurement accuracy of a non-metric camera; this directly affects the accuracy of a traffic accident scene investigation.

In this paper, an improved 2D-DLT measurement method is proposed based on a composite of small calibration objects, thus addressing the difficulty in arranging large calibration objects and the low measuring accuracy of small calibration objects. In this method, several small calibration objects are placed around the traffic accident scene. Because of the constancy of the relative positions and the coplanar relationship between the small calibration objects, the local coordinate system of each small calibration object is transformed into the coordinate system of the composite calibration object. Then, a minimum reprojection error objective function is established, and nonlinear optimization methods are used to rectify the image.

The paper is organized as follows. First, the camera model is introduced, and the synthesized calibration principle using composite small calibration objects is proposed. Using this principle, the improved 2D-DLT method is established. Then, using a non-metric consumer-grade camera and a self-developed calibration object, several field experiments and specific case analyses are performed to demonstrate the feasibility of this method. Finally, discussions are presented and conclusions are drawn.

## Methods

### The Camera Model

**Fig 1:**
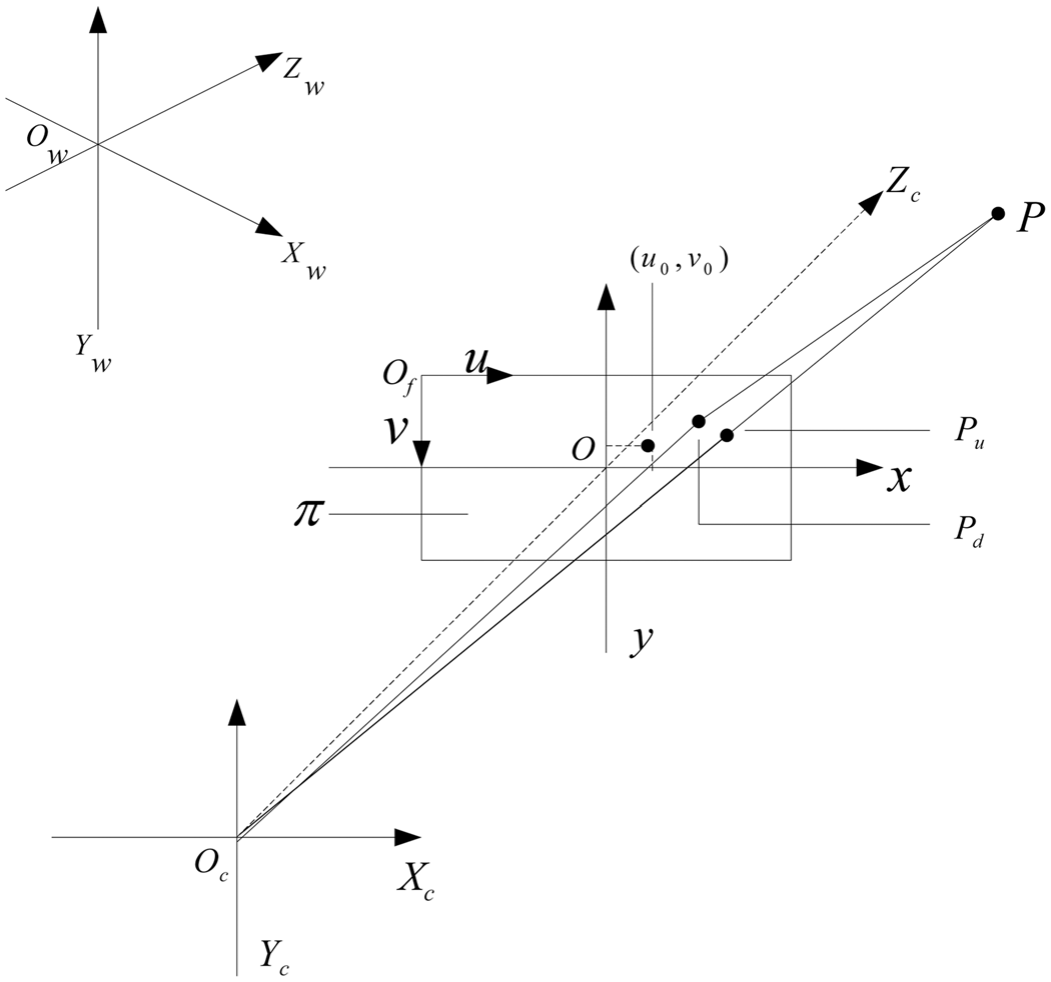
Geometry model of camera imaging.

The perspective projection model [20] is one of the camera models used in traffic accident photogrammetry; the coordinate transformation between the object space and the image space is shown in Fig 1.

In Fig 1, *π* is the camera image plane, *O*_*f*_ *uv* is the pixel coordinate system, *Oxy* is the image plane coordinate system, *O*_*c*_ *X*_*c*_ *Y*_*c*_ *Z*_*c*_ is the camera coordinate system, and *O*_*w*_ *X*_*w*_ *Y*_*w*_ *Z*_*w*_ is the word coordinate system. The camera's optical center *O*_*c*_ is the origin of the camera coordinate system, with shaft axes *X*_*c*_, *Y*_*c*_ respectively parallel to axes *x* and *y* in *Oxy*, and *Z*_*c*_ overlapping the optical axis of the camera. The origin *O* of the image plane coordinate system is on the optical axis, and is called the main point. Its pixel coordinate is (*u*_*o*_, *v*_*o*_) in *O*_*f*_ *uv*, which will be the offset in the imaging process. The distance *O*_*c*_ *O* is the camera's focal length *f*. The origin *O*_*f*_ in the pixel coordinate system is located in the upper left corner of the image, with shaft axes *u* and *v* respectively parallel to axes *x* and *y* in *Oxy*.

Given a space point *P*, *P*_*w*_ = [*X*_*w*_ *Y*_*w*_ *Z*_*w*_ 1]^T^ are the homogeneous coordinates in *O*_*w*_ *X*_*w*_ *Y*_*w*_ *Z*_*w*_, and *P*_*c*_ = [*X*_*c*_ *Y*_*c*_ *Z*_*c*_ 1]^T^ are the homogeneous coordinates in *O*_*c*_ *X*_*c*_ *Y*_*c*_ *Z*_*c*_, whose coordinate unit is the metric system. Let *P*_*u*_ be the undistorted image point of the homogeneous coordinates in *Oxy*. *P*_*d*_ is the actual distorted image point of the homogeneous coordinates in the imaging process, and *u*_*f*_ = [*u v* 1]^T^ are the homogeneous coordinates in *O*_*f*_ *uv*, whose coordinate unit is the pixel system.

In the undistorted image, the camera imaging model can be represented by the following formula:

$$\lambda u_{f}=\lambda \left[\begin{array}{c} u\\ v\\ 1 \end{array}\right]=\left[\begin{array}{cccc} a_{x} & c & u_{o} & 0\\ 0 & a_{y} & v_{o} & 0\\ 0 & 0 & 1 & 0 \end{array}\right]\left[\begin{array}{cccc} r_{11} & r_{21} & r_{31} & t_{x}\\ r_{12} & r_{22} & r_{32} & t_{y}\\ r_{13} & r_{23} & r_{33} & t_{z}\\ 0 & 0 & 0 & 1 \end{array}\right]\left[\begin{array}{c} X_{w}\\ Y_{w}\\ Z_{w}\\ 1 \end{array}\right]=\left[\begin{array}{cc} A & 0 \end{array}\right]\left[\begin{array}{cc} R & T\\ 0^{T} & 1 \end{array}\right]P_{w}$$ (1)

where *λ* is an arbitrary proportion coefficient. *A* denotes the matrix for the inner orientation of the camera parameters, which relates to parameters *a*_*x*_, *a*_*y*_, *c*, *u*_*o*_, *v*_*o*_, and describes the parameters of the relative position between the photograph center and the photos, and represents the camera's own characteristics. *R* and *T*, respectively, denote the rotation and translation matrices from the world coordinate system to the camera coordinate system; these are called the exterior orientation parameters of the camera, and represent the parameters of the photography beam in the spatial position and the posture when photographing.

In actual image processing, because of the camera lens distortion, the image point deviates from the ideal location, as shown in Fig 1. The distortion point *P*_*d*_ deviates from the non-distortion point *P*_*u*_, which affects the accuracy of the photogrammetry [21]. Here, the effects of the radial distortion and the tangential distortion of the image processing are considered simultaneously. The relationship between the pixel coordinates (*u*, *v*) of distortion point *P*_*d*_ (*x*_*d*_, *y*_*d*_) and the coordinates of *P*_*u*_ (*x*_*u*_, *y*_*u*_) can be expressed as

$$\{\begin{array}{c} x_{u}=u-u_{o}+\left(u-u_{o}\right)\left(k_{1}r^{2}+k_{2}r^{4}\right)+\left(r^{2}+2{\left(u-u_{o}\right)}^{2}\right)p_{1}+2\left(u-u_{o}\right)\left(v-v_{o}\right)p_{2}\\ y_{u}=v-v_{o}+\left(v-v_{o}\right)\left(k_{1}r^{2}+k_{2}r^{4}\right)+2\left(u-u_{o}\right)\left(v-v_{o}\right)p_{1}+\left(r^{2}+2{\left(v-v_{o}\right)}^{2}\right)p_{2} \end{array}$$ (2)

where *r* ^2^ = (*u−u*_*o*_)^2^ + (*v−v*_*o*_)^2^ is the radial distance from an imaging point to the optical axis; *k*_1_ and *k*_2_ respectively denote the first- and second-order radial distortion coefficients; *p*_1_ and *p*_2_ respectively denote the first- and second-order tangential distortion coefficients.

### Principle of Composite Small Calibration Objects

When using a non-metric camera and a single small calibration object for traffic accident scene photogrammetry, the transformation relationship between the small calibration object’s coordinate system and the camera coordinate system can be obtained as

$$P_{c}=\left[\begin{array}{cc} R & T\\ 0^{T} & 1 \end{array}\right]P_{w}$$ (3)

Feature point *P*_*i*_ has homogeneous coordinates *P*_*wi*_ when it is located on a small calibration object *i*. Its homogeneous coordinates are *P*_*wj*_ for a small calibration object *j*. The homogeneous coordinates in the camera coordinate system of that point, respectively, can be represented as

$$P_{ci}=\left[\begin{array}{cc} R_{i} & T_{i}\\ 0^{T} & 1 \end{array}\right]P_{wi}$$ (4)

$$P_{cj}=\left[\begin{array}{cc} R_{j} & T_{j}\\ 0^{T} & 1 \end{array}\right]P_{wj}$$ (5)

Clearly, *P*_*ci*_ = *P*_*cj*_. The coordinate system transformation relationship between small calibration objects *i* and *j* can be deduced by simultaneously solving the above equations.

$$\left[\begin{array}{cc} R_{i} & T_{i}\\ 0^{T} & 1 \end{array}\right]P_{wi}=\left[\begin{array}{cc} R_{j} & T_{j}\\ 0^{T} & 1 \end{array}\right]P_{wj}$$ (6)

The above equation can be simplified as

$$P_{wi}={\left[\begin{array}{cc} R_{i} & T_{i}\\ 0^{T} & 1 \end{array}\right]}^{-1}\left[\begin{array}{cc} R_{j} & T_{j}\\ 0^{T} & 1 \end{array}\right]P_{wj}=R_{i}^{-1}R_{j}P_{wj}+R_{i}^{-1}\left(T_{j}-T_{i}\right)$$ (7)

Let $RT_{i}=\left[\begin{array}{cc} R_{i} & T_{i}\\ 0^{T} & 1 \end{array}\right]$ and $RT_{j}=\left[\begin{array}{cc} R_{j} & T_{j}\\ 0^{T} & 1 \end{array}\right]$ ; the equation can be abbreviated as

$$P_{wi}=RT_{i}{}^{-1}RT_{j}P_{wj}$$ (8)

**Fig 2:**
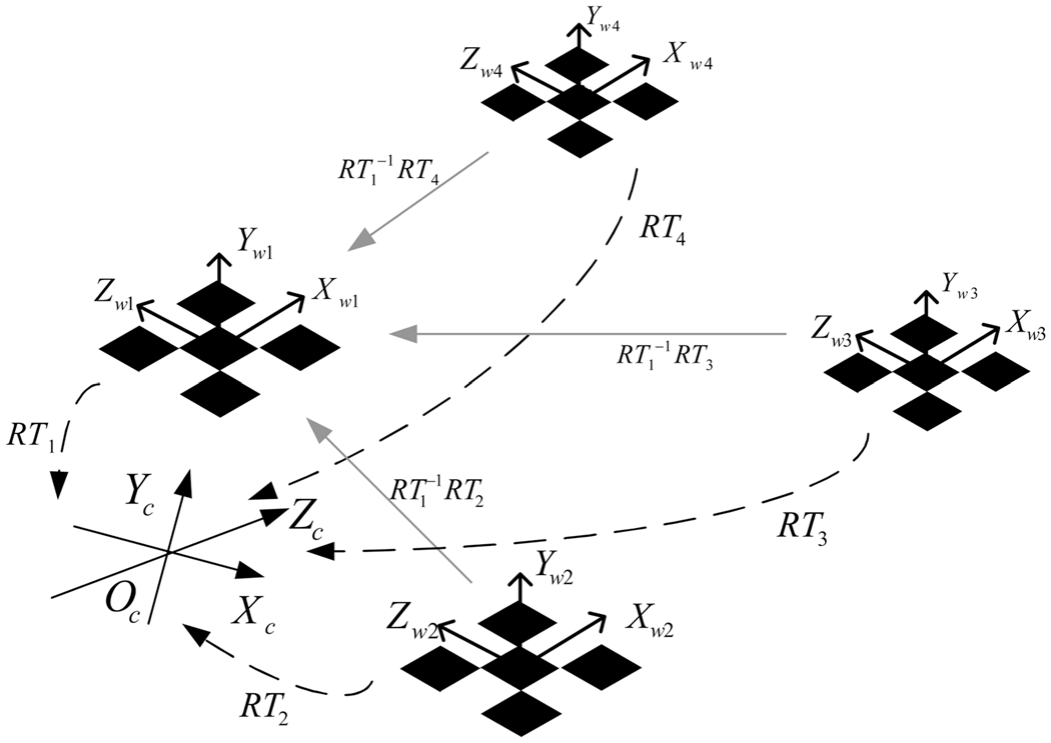
Scheme of a synthetic calibration object.

Using the above formula, a large calibration object can be composed of a number of small calibration objects, as shown in Fig 2. The key is to calculate the exterior orientation parameters *RT*_*i*_ of each small calibration object relative to the camera. Then, the coordinates of each small calibration object on the composite calibration object can be calculated by Eq (8), effectively replacing the small calibration objects and expanding the measurement range.

In order to calculate *RT*_*i*_ of each small calibration object, the homographic matrix *H* that contacts the camera plane and the calibration object plane should be calculated first. Then, the camera parameters are calculated, i.e., *RT*_*i*_ is determined. Finally, the small calibration objects are composited completely.

#### Calculate homographic matrix H

As shown in Fig 2, the calibration object in 2D photogrammetry is planar, which is the *Y*_*w*_ = 0 plane in the world coordinate system. Let *r*_*i*_ denote the column vectors of matrix *R*; then, Eq (1) can be transformed into

$$\lambda u_{f}=\lambda \left[\begin{array}{c} u\\ v\\ 1 \end{array}\right]=\left[\begin{array}{cc} A & 0 \end{array}\right]\left[\begin{array}{cccc} r_{1} & r_{2} & r_{3} & T\\ 0 & 0 & 0 & 1 \end{array}\right]\left[\begin{array}{c} X_{w}\\ 0\\ Z_{w}\\ 1 \end{array}\right]=A\left[\begin{array}{ccc} r_{1} & r_{3} & T \end{array}\right]\left[\begin{array}{c} X_{w}\\ Z_{w}\\ 1 \end{array}\right]=HP_{w}$$ (9)

where *H* = [*h*_1_ *h*_2_ *h*_3_] is the homographic matrix, which can be calculated according to the 2D-DLT method. This method uses a plane calibration object in which the point’s coordinates are known and will take at least four points [15, 22].

#### Solve the camera parameters

According to the orthogonality characteristics of the rotation matrix *R*, the two parameter constraint conditions are

$$\{\begin{array}{c} r_{1}^{T}r_{3}=h_{1}^{T}A^{-T}A^{-1}h_{2}=0\\ r_{1}^{T}r_{1}=h_{1}^{T}A^{-T}A^{-1}h_{1}=h_{2}^{T}A^{-T}A^{-1}h_{2}=r_{3}^{T}r_{3} \end{array}$$ (10)

According to Zhang [23], we obtain *A* ^-1^, *A* using matrix decomposition.

After solving the exterior orientation parameters of each image, *RT*_*i*_ can be calculated.

$$r_{1}=A^{-1}h_{1};r_{3}=A^{-1}h_{2};r_{2}=r_{1}\times r_{3};T=A^{-1}h_{3}$$ (11)

#### Establish the composite calibration object’s point coordinates

The key to establishing a composite calibration object is to determine the positional relationship between each small calibration object coordinate system and the composite calibration object coordinate system. The coordinates of a point on the photo are the same as those on the camera coordinate system for the media.

Assume that we choose the *jth* small calibration object’s coordinate system for the composite calibration object. Feature point *P*_*i*_ on the *ith* calibration object’s coordinate system has homogeneous coordinates of *P*_*wi*_, whereas *P*_*wj*_ is on the composite calibration object’s coordinate system. The homogeneous coordinates of this point are the same as in the camera coordinate system. From Eqs (7) and (8), the transformation of the relationship between the coordinate system of the *ith* calibration object and the composite calibration object is

$$P_{wj}=RT_{j}^{-1}RT_{i}P_{wi}={\left[\begin{array}{cc} R_{j} & T_{j}\\ 0^{T} & 1 \end{array}\right]}^{-1}\left[\begin{array}{cc} R_{i} & T_{i}\\ 0^{T} & 1 \end{array}\right]P_{wi}=R_{j}^{-1}R_{i}P_{wi}+R_{j}^{-1}\left(T_{i}-T_{j}\right)$$ (12)

Depending on the characteristics of the traffic accident scene, multiple small calibration objects can be placed near the scene of the accident. This method can composite the small calibration objects, and form a calibration area as large as necessary.

### Improved 2D-DLT Method

After determining the coordinates of a calibration point in the composite calibration object, the entire region of interest in the accident scene can be covered. Based on a plane calibration object, the 2D-DLT photogrammetry method can be used for accident scene investigation. This can correctly reflect the perspective projection relationship between the space and image planes of the entire scene.

**Fig 3:**
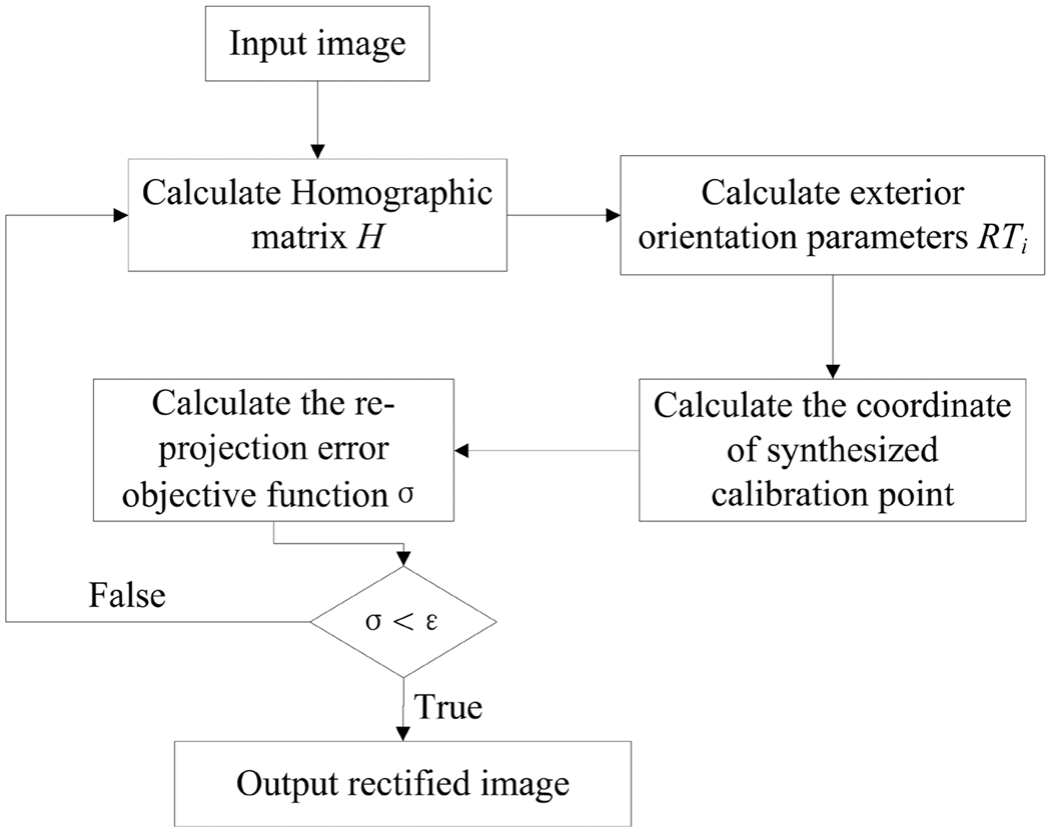
Flow chart of the improved 2D-DLT method.

In order to improve the accuracy of the measurement, the effect of lens distortion is considered, and a minimum reprojection error objective function is established as

$$\sigma =\min\sum_{i=1}^{n}{\left(q_{i}-q\left(A,k_{1},k_{2},p_{1},p_{2},P_{wi}\right)\right)}^{2}$$ (13)

where *n* is the number of calibration points involved in the calculation, *q*_*i*_ denotes the pixel coordinates of the *ith* feature point on the image plane, and *P*_*wi*_ denotes the composite calibration object coordinates of the *ith* feature point. The pixel coordinates *q* (*A*, *k*_1_, *k*_2_, *p*_1_, *p*_2_, *P*_*wi*_) of feature point *P*_*wi*_ are obtained by camera model relations. The values of parameters *A*, *k*_1_, *k*_2_, *p*_1_, *p*_2_, and *P*_*wi*_ that minimize the objective function *σ* are the optimal solution, through the nonlinear optimization method. The process of the improved 2D-DLT method is shown in Fig 3.

## Experimental Verification

### Preliminary Experiments

**Fig 4:**
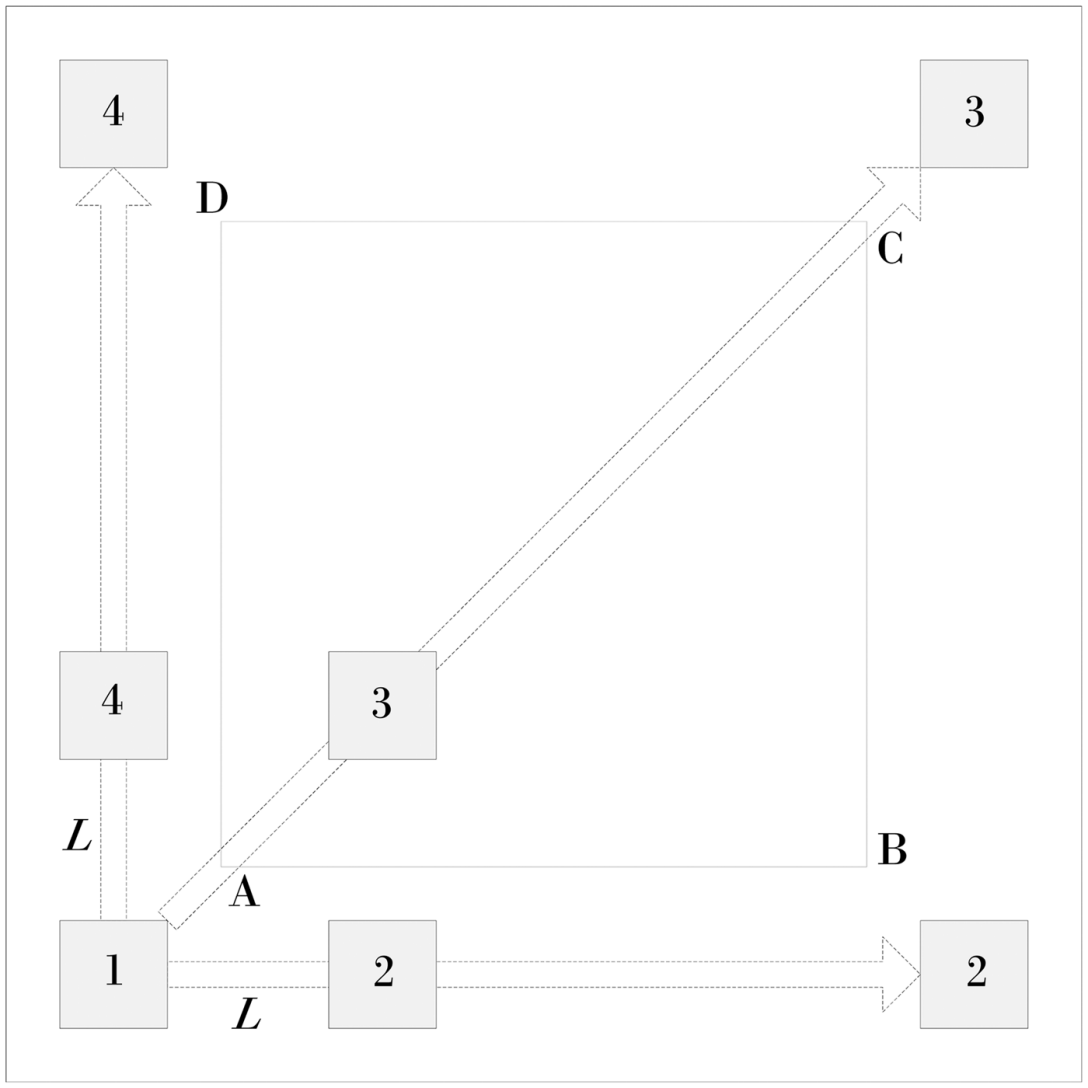
Sketch map of the experimental site.

According to the above model, a set of experiments was designed to examine the effect of the area covered by the composite calibration object, and the size of the small calibration object on the measurement results. A non-metric consumer-grade camera, i.e., Nikon D3200, was used in the experiments. The image resolution was 3008 × 2000 pixels; the photography distance was approximately 3.0 m. The measurement experiment site was 4.0 × 4.0 m. The composite calibration object was a square area that consisted of four small calibration objects. The number of feature points on each small calibration object was 4 × 4. The horizontal and vertical spacing of the feature point was 0.1 m, as shown in Fig 4.

In Fig 4, the composite calibration object is made up of four small calibration objects labeled 1, 2, 3, and 4; L is the length of the composite calibration object; the side length of quadrilateral *ABCD*, 3.0 m, is the measurement distance.

By positioning different small calibration objects, the 2D image rectification was conducted on site, followed by surveying and mapping. Finally, the data was analyzed to determine the relative error of the measuring distance.

#### Impact of composite calibration object region on measurement results

The most important feature of a composite calibration object is the flexibility in placing the small calibration objects, which can form composite calibration objects of different sizes depending on the scene of the accident. The following analysis describes the effect of the size of the composite calibration object on the photogrammetry results; the size and quantity of small calibration objects remained constant.

**Fig 5:**
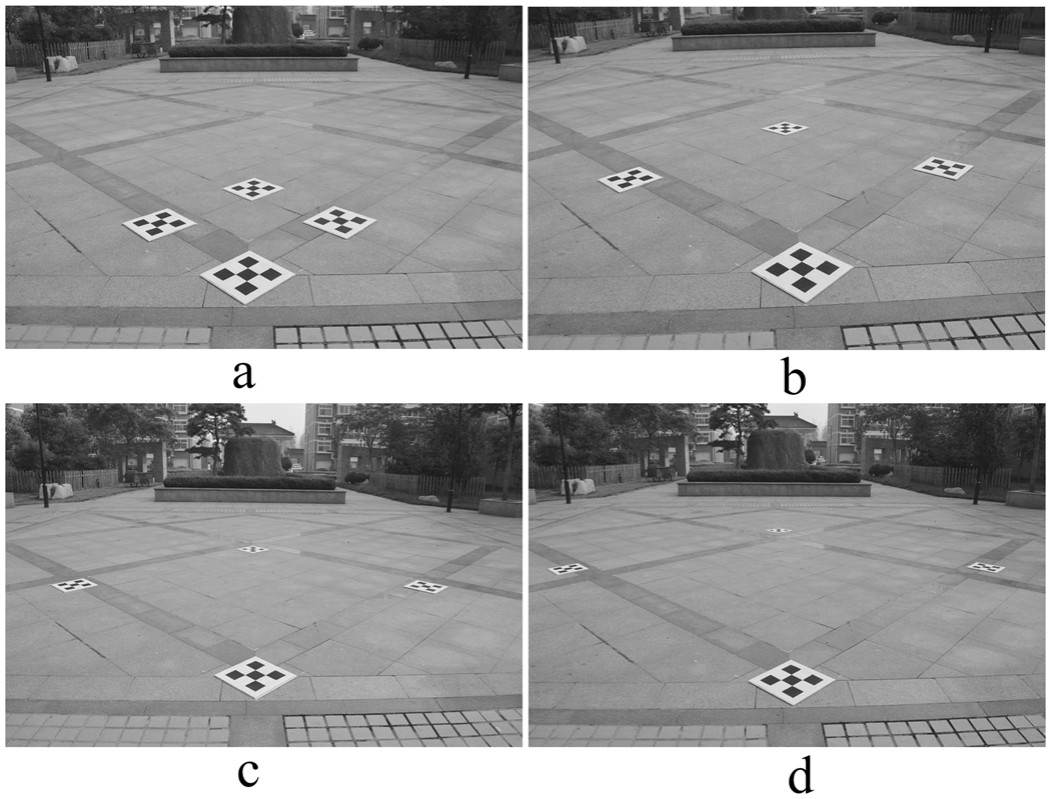
Photo of experimental site. Four field images were taken as the length *L* of the composite calibration object varied from 1.0 m to 4.0 m. (a) 1.0 m, (b) 2.0 m, (c) 3.0 m, (d) 4.0 m.

**Fig 6:**
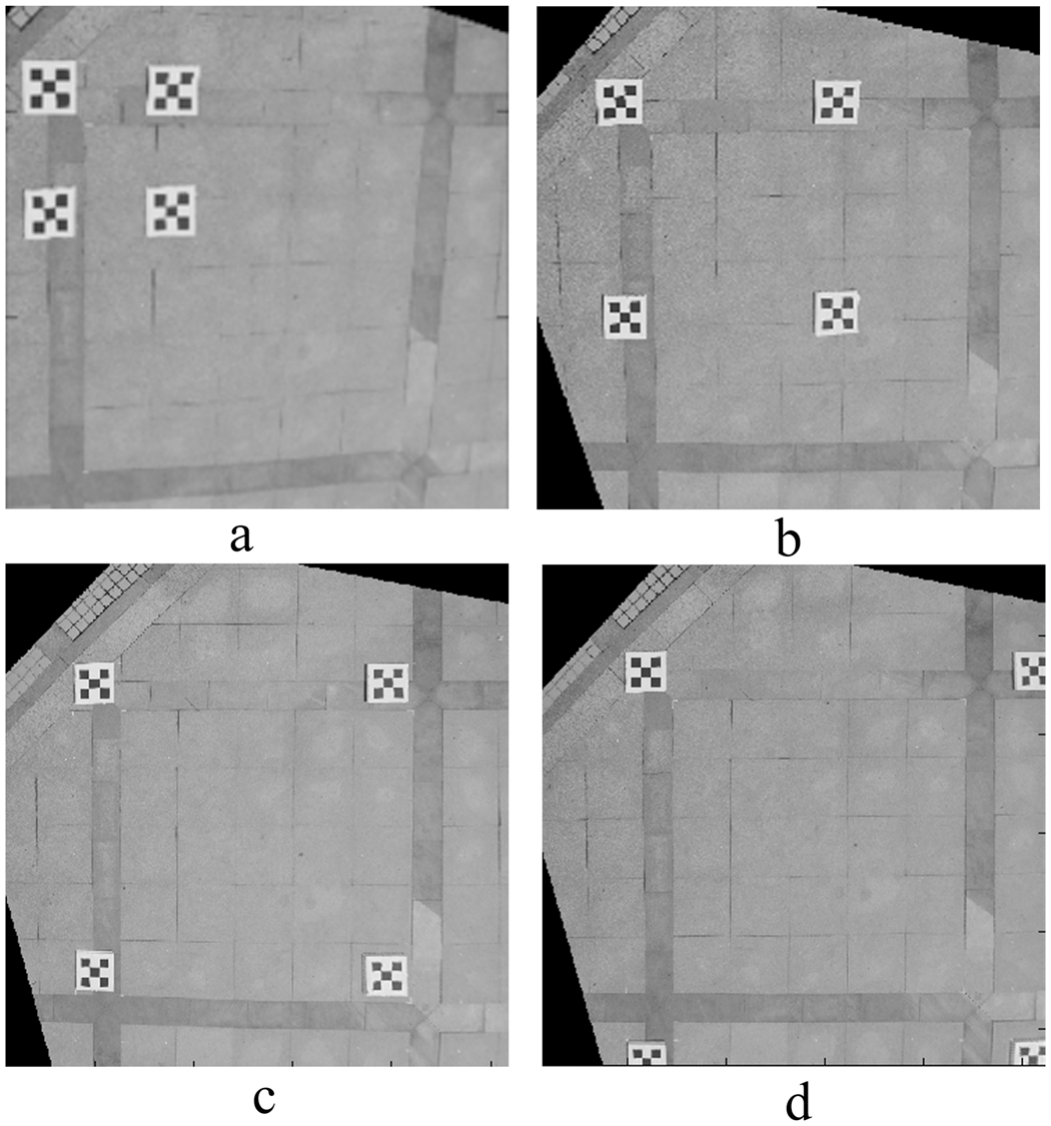
Rectified image of experimental site. (a), (b), (c), (d) are the rectified images corresponding to Fig 5.

The length *L* of the composite calibration object varied from 1.0 m to 4.0 m, increasing each time by 1.0 m; four field images were taken, as shown in Fig 5. The image rectified by the proposed correction algorithm is shown in Fig 6. The relative measurement error of each side of quadrilateral ABCD is shown in Table 1.

**Table 1:** Relative error of the length of each side of the quadrangle.

*L* (m)	*AB* (%)	*BC* (%)	*CD* (%)	*DA* (%)	AVG* (%)
**1.0**	13.49	20.64	18.94	15.96	17.26
**2.0**	3.62	5.75	3.85	1.74	3.74
**3.0**	4.18	2.08	0.89	1.41	2.14
**4.0**	1.01	1.42	0.31	1.06	0.95

*AB*, *BC*, *CD*, and *DA* denote the relative measuring error of each side of quadrilateral ABCD. *L* is the length of the composite calibration object.

*The average value

From Fig 6 and Table 1, we observe that the greater the area of the composite calibration object, the better are the photography measurement results, which further confirms the effectiveness of a large composite calibration object created by closely linking each small calibration object with *RT*_*i*_. Therefore, when conducting a traffic accident scene investigation, small calibration objects should be placed around the scene, forming a large composite calibration object to reduce the relative photogrammetric error.

#### Effect of small calibration object size on calibration results

**Fig 7:**
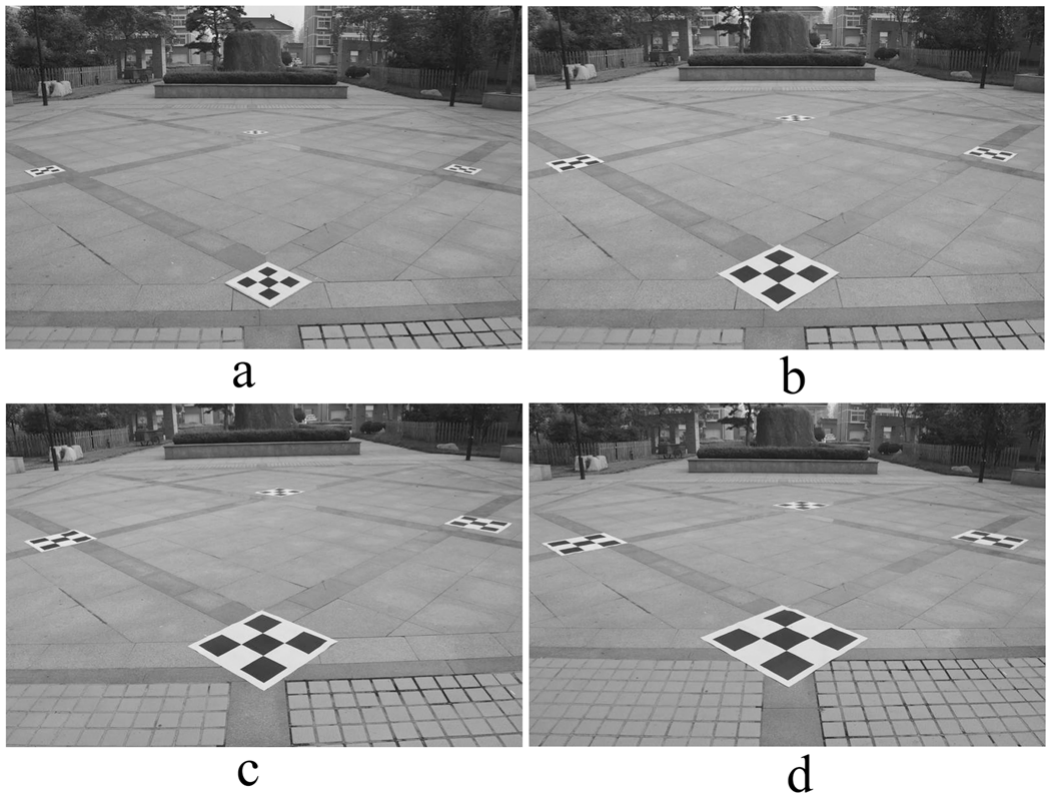
Photo of experimental site. The size of the composite calibration object was 4.0 m × 4.0 m; the horizontal and vertical spacing Δ*l* of feature points on the small calibration object varied from 0.1 m to 0.25 m. (a) 0.1 m, (b) 0.15 m, (c) 0.2 m, (d) 0.25 m.

**Fig 8:**
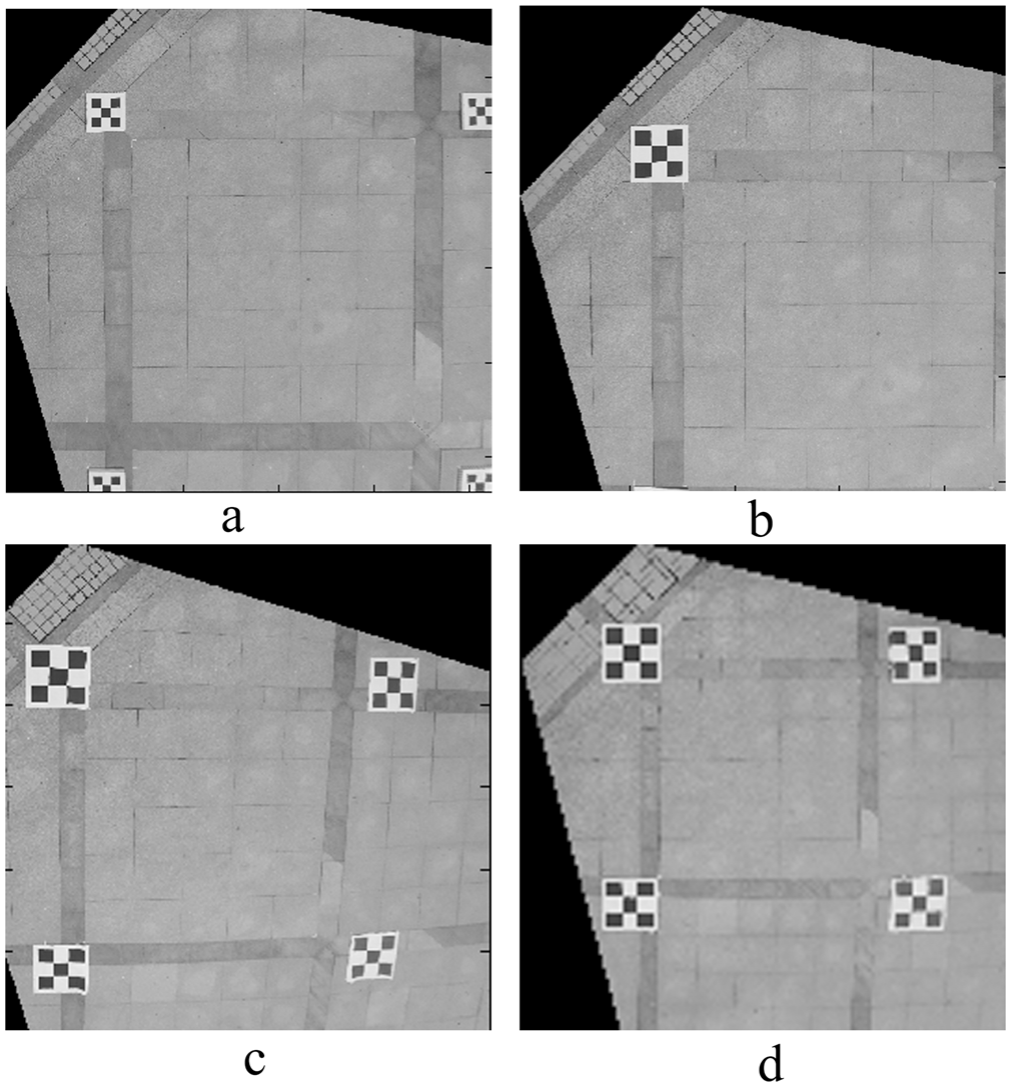
Rectified image of experimental site. (a), (b), (c), (d) are the rectified images corresponding to Fig 7.

The size of the composite calibration object was 4.0 × 4.0 m. The horizontal and vertical spacing Δ*l* of feature points on the small calibration object varied from 0.1 m to 0.25 m, increasing each time by 0.05 m; four field images were taken, as shown in Fig 7. The image rectified by the proposed correction algorithm is shown in Fig 8. The relative measuring error of each side of quadrilateral ABCD is shown in Table 2.

**Table 2:** Relative error of the length of each side of the quadrangle.

Δ*l* (m)	*AB* (%)	*BC* (%)	*CD* (%)	*DA* (%)	AVG* (%)
**0.10**	1.01	1.42	0.31	1.06	0.95
**0.15**	1.16	2.83	0.67	2.10	1.69
**0.20**	1.17	1.28	0.65	1.37	1.12
**0.25**	0.96	0.95	3.13	1.07	0.82

*AB*, *BC*, *CD*, and *DA* denote the relative measuring error of each side of quadrilateral ABCD. Δ*l* is the length of the horizontal and vertical spacing of feature points on the small calibration object.

*The average value

From Fig 8 and Table 2, with a constant composite calibration object area, as long as the small calibration object is legible, the photogrammetry results are the same, despite the increase in the small calibration object’s size.

### Comparative Experiment

**Fig 9:**
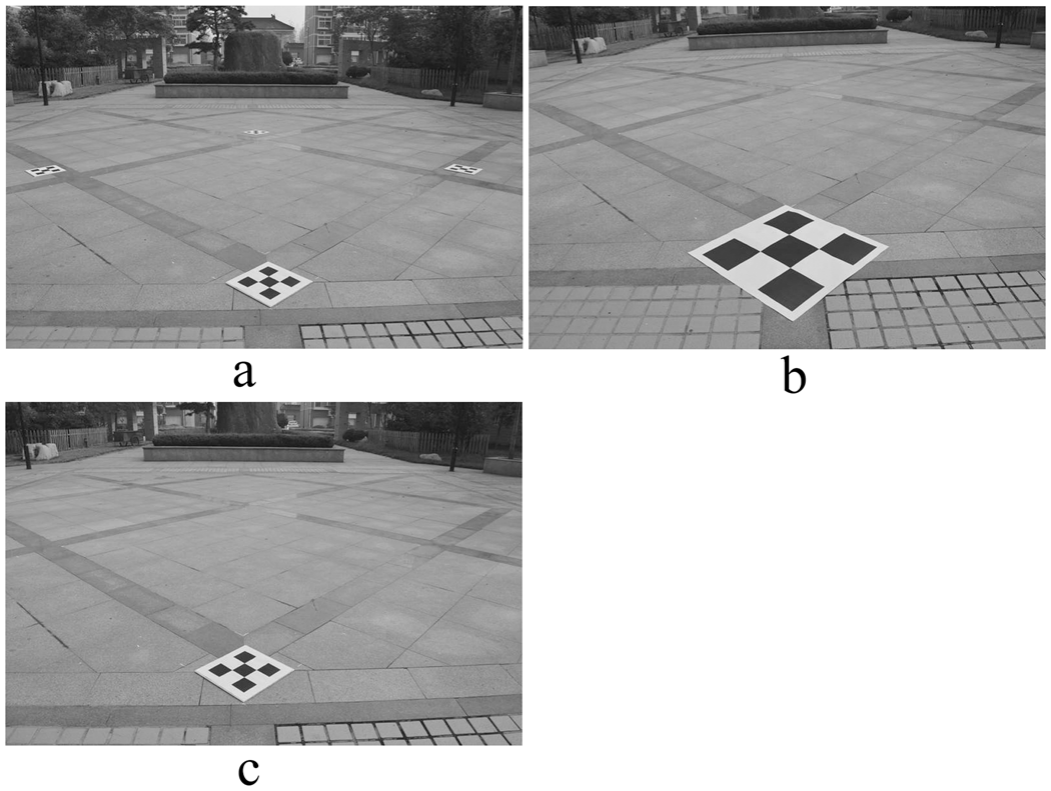
Photo of experimental site. (a) Four small calibration objects were distributed at the four corners of the experiment site; the horizontal and vertical spacing of the feature points was 0.1 m. (b) Using a single large calibration object; the horizontal and vertical spacing of the feature points was 0.25 m. (c) Using a single small calibration object that is the same as each small composite calibration object.

**Fig 10:**
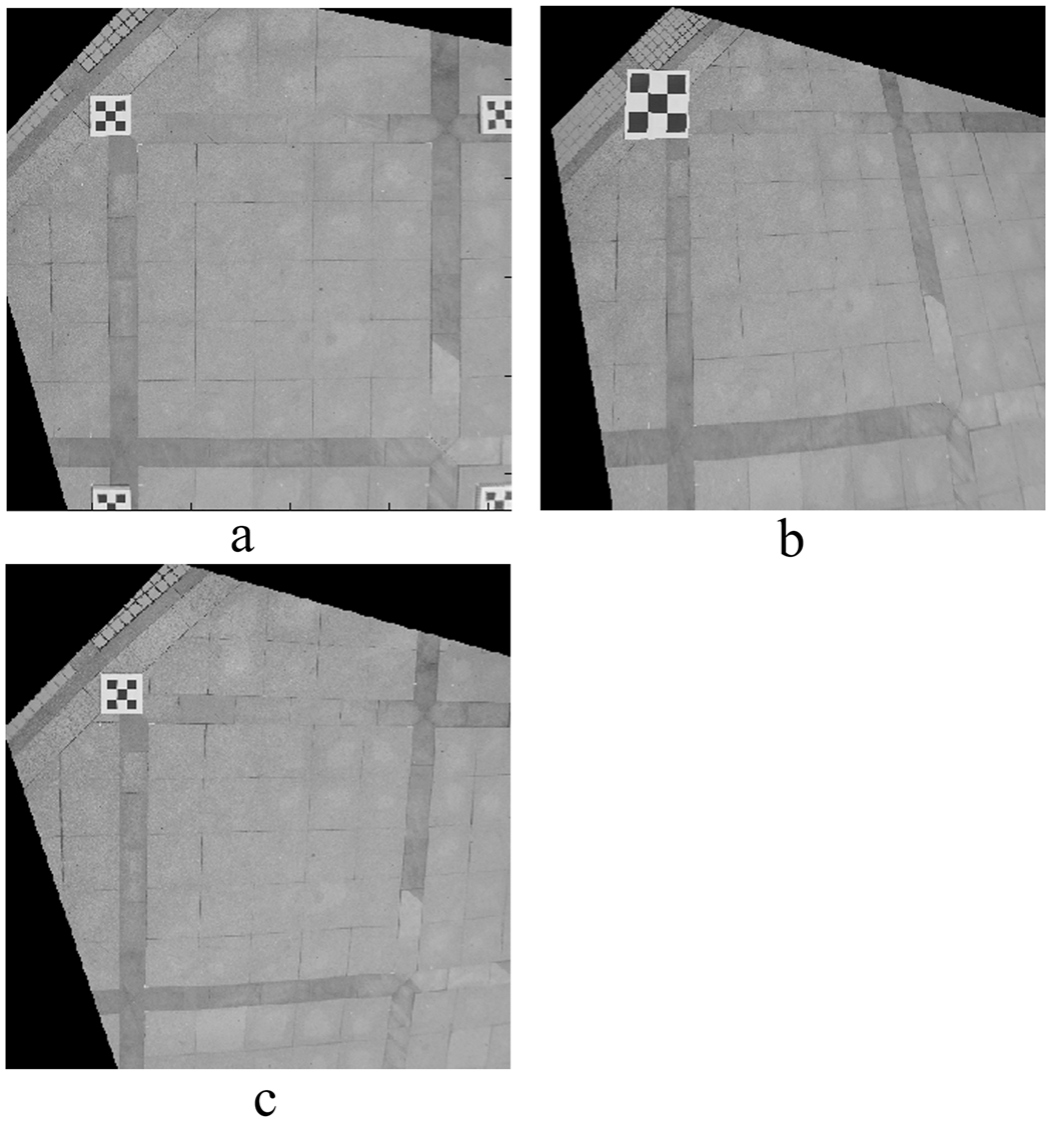
Rectified image of experimental site. (a), (b), (c) are the rectified images corresponding to Fig 9.

In order to verify the superiority of a composite calibration object for photogrammetry, three comparative experiments were conducted. The first group used a composite calibration object according to the proposed method. Four small calibration objects were distributed at the four corners of the experiment site; the horizontal and vertical spacing of the feature points was 0.1 m. The second group used a single large calibration object; the horizontal and vertical spacing of the feature point was 0.25 m. The third group used a single small calibration object that was the size of a small composite calibration object. Three field images were taken, as shown in Fig 9. The image, rectified using the method described in this article, is shown in Fig 10. The relative measuring error of each side of quadrilateral ABCD is shown in Table 3.

**Table 3:** Relative error of the length of each side of the quadrangle.

NO.	*AB* (%)	*BC* (%)	*CD* (%)	*DA* (%)	AVG* (%)
**1**	1.01	1.42	0.31	1.06	0.95
**2**	25.30	6.61	18.33	12.46	15.68
**3**	10.07	14.80	15.51	9.30	12.42

*AB*, *BC*, *CD*, and *DA* denote the relative measuring error of each side of quadrilateral ABCD. No. denotes the three groups of experiments.

*The average value

From Fig 10 and Table 3, the relative error using the composite calibration object for photogrammetry was minimal, which is far better than the results using a single calibration object. When using a single calibration object, the size had no significant influence on the measurement results. The measuring accuracy was greatly influenced by the location of the calibration object.

A comprehensive analysis of the three groups of experimental results showed that the composite calibration object provided a wide field range, with mutual constraint relationships between the feature points. Through these feature points, the camera’s internal parameters well presented the representative perspective transformation relation between the camera and the scene plane. A small calibration object provided a smaller range of feature point distribution, which resulted in lower accuracy in camera model solving, and a larger photography measurement error.

## Results and Discussion

**Fig 11:**
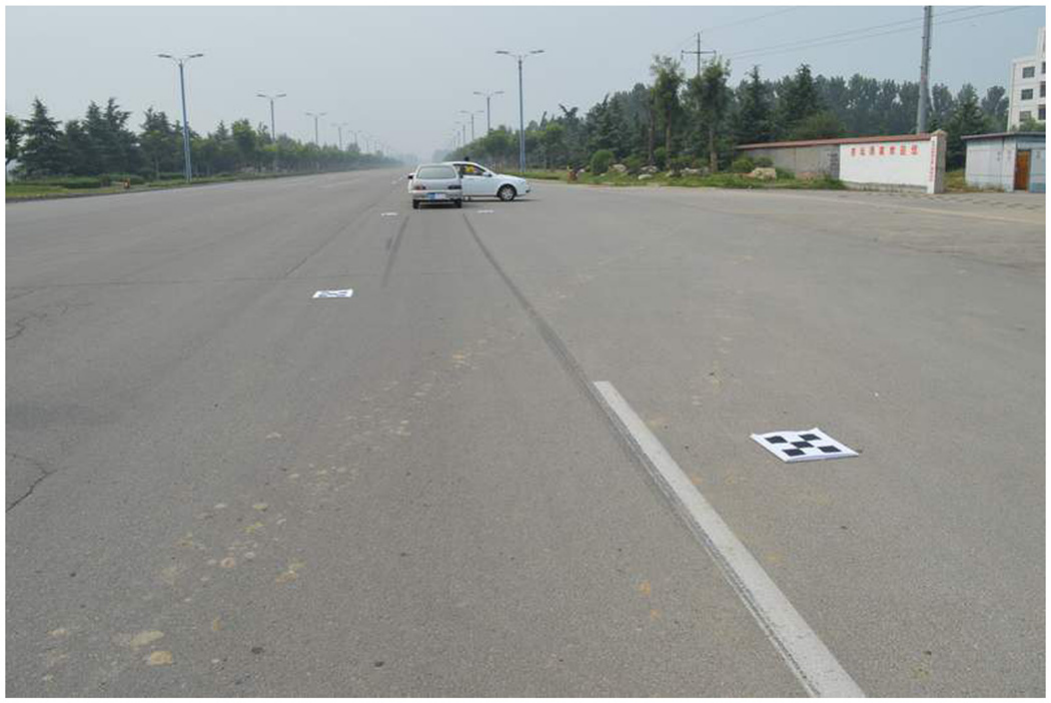
Photo of traffic accident site.

Fig 11 shows a traffic accident that occurred in 2014; the accident vehicles left braking traces on the road. The composite calibration object consisted of four small calibration objects that were placed around the area of the accident scene. The number of feature points on each small calibration object was 4 × 4, and the horizontal and vertical spacing of the feature points was 0.1 m. The image resolution was 3008 × 2000 pixels. The main goal of this case was to measure the vehicle stop position and the braking trace length, in order to analyze the braking vehicle’s original speed, and other information.

**Fig 12:**
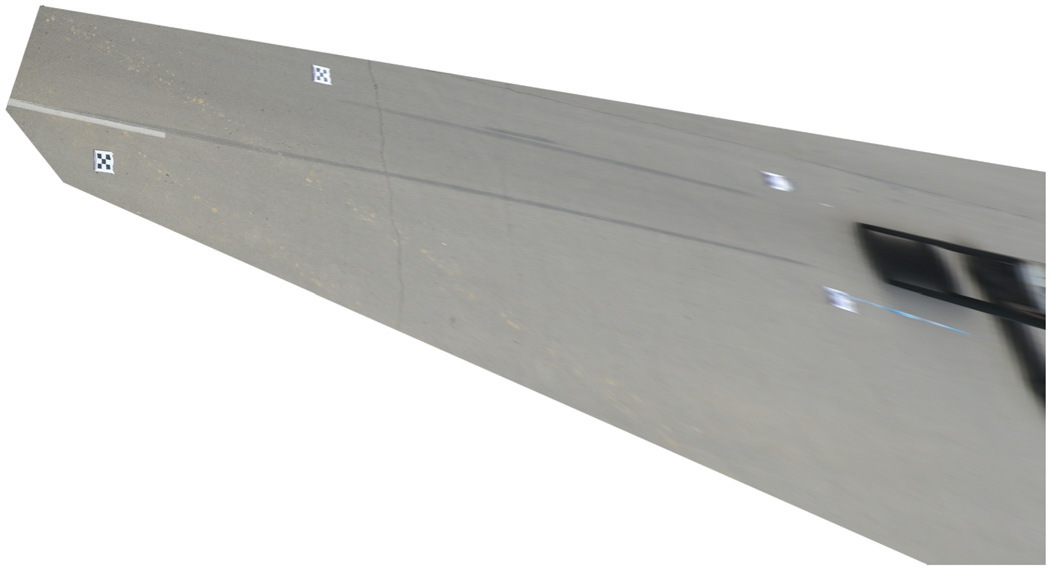
Rectified image of traffic accident site.

In order to facilitate the comparative analysis, the traditional manual measurement method and the photogrammetry method using the proposed composite calibration object were conducted simultaneously. The image rectified by the proposed correction algorithm is shown in Fig 12.

The main factors are reflected in the rectified image of the scene of the accident. The relative measuring errors of the traditional hands-on method and the proposed method are shown in Table 4.

**Table 4:** Measurement comparison between the hands-on method and the proposed method.

Data description	Length of left braking trace	Length of right braking trace
**Hands-on method (m)**	12.10	22.00
**Proposed method (m)**	12.61	22.43
**Relative error (%)**	5.04	1.95

**Fig 13:**
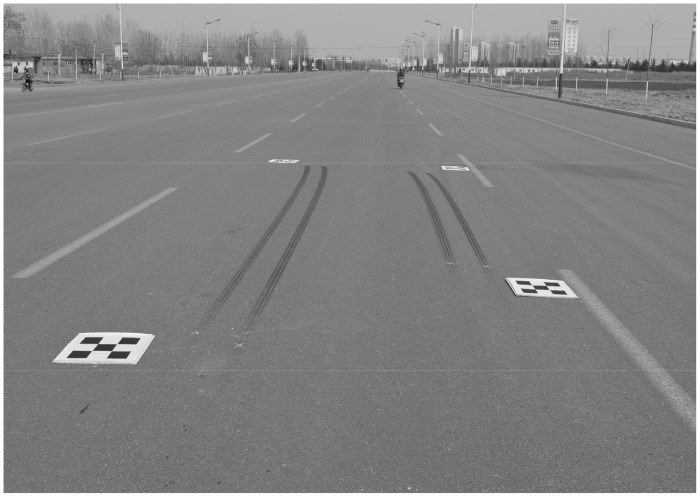
Photo of traffic accident site.

**Fig 14:**
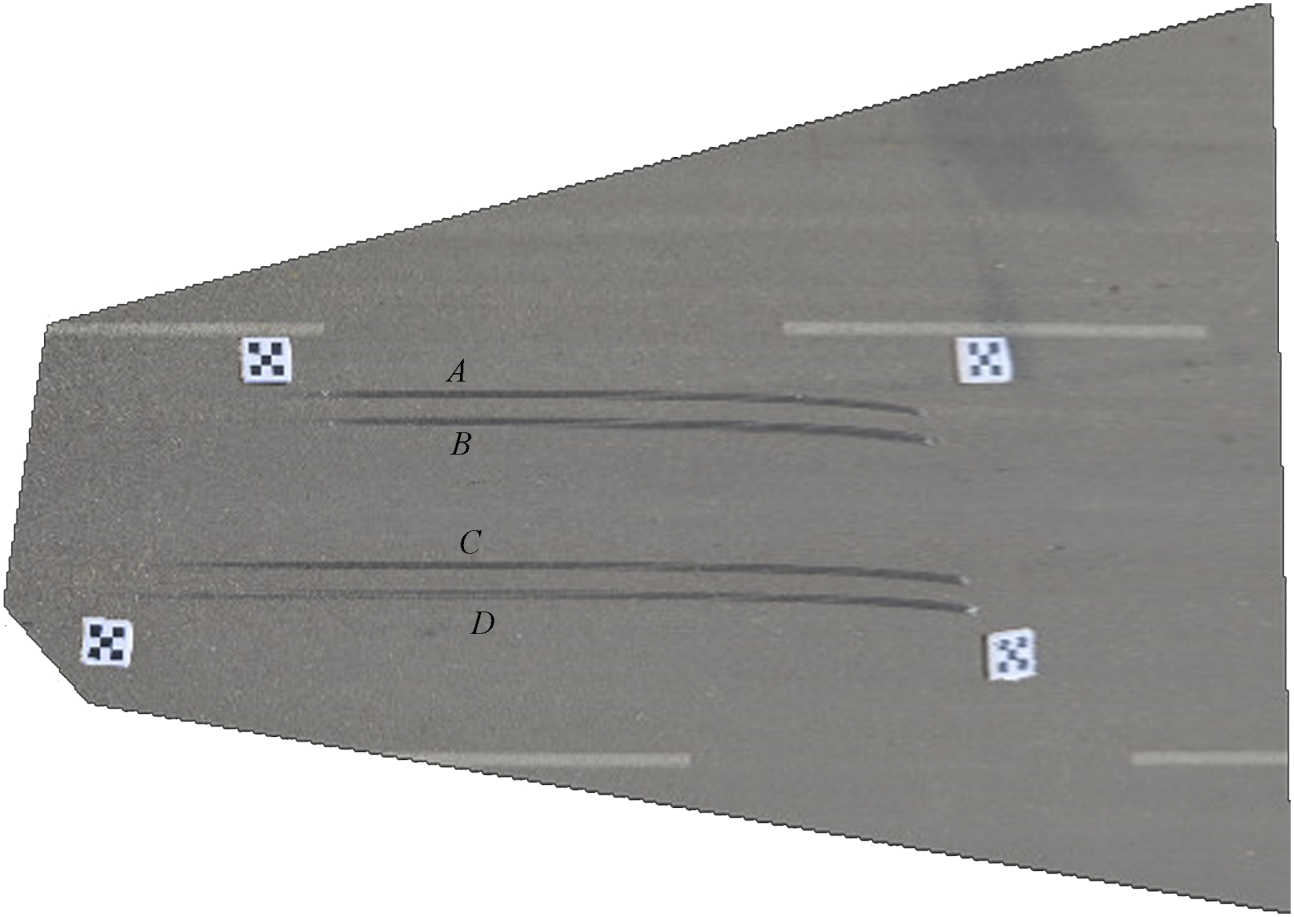
Rectified image of traffic accident site.

Fig 13 shows another forensic photograph. We use the same method to analyze this traffic accident scene. The rectified image is shown in Fig 14. The accuracy of the braking trace reconstructed using the proposed method can be evaluated by comparing with the traditional hands-on method. Their relative measuring errors are shown in Table 5.

**Table 5:** Measurement comparison between the hands-on method and the proposed method.

Data description	Length of braking trace *A*	Length of braking trace *B*	Length of braking trace *C*	Length of braking trace *D*
**Hands-on method (m)**	7.40	7.50	5.30	5.31
**Proposed method (m)**	7.25	7.28	5.19	5.21
**Relative error (%)**	2.02	2.93	2.07	1.88

*A*, *B*, *C*, and *D* denote the breaking trace mark in Fig 14.

From Tables 4 and 5, the maximum relative errors are 5.04% and 2.93%, which is well within the accuracy acceptable for traffic accident scene investigation.

Using the method of composite small calibration objects for photogrammetry, small calibration objects can be flexibly arranged at the scene of the accident. In order to meet the measurement accuracy requirements, the small calibration object must be clearly visible. Otherwise, the size of small calibration object must be increased to fit the scene. After calibrating the entire site area, the scene of the accident can be measured by the improved 2D-DLT method, which can avoid the influence of artificial factors. To a certain extent, this method can improve the accuracy of traffic accident scene investigation. The feasibility of using a composite calibration object for traffic accident scene investigation is demonstrated.

## Conclusions

When using a non-metric consumer-grade camera for photogrammetry, the calibration object should cover the entire space. The ratio of the area that the calibration object occupies in the accident site and the location of the calibration object greatly affect the measurement accuracy. Because traffic accident scenes can be up to dozens of meters, it is generally not realistic to produce a calibration object sufficiently large for the scene. An improved 2D-DLT measurement method was proposed, based on a composite small calibration object. At a traffic accident scene, multiple small calibration objects were flexibly placed to include a large region of interest, thereby overcoming the limitations of a single small calibration object.

By maintaining the relative positions and coplanar relationship between small calibration objects, the local coordinate system of each small calibration object was transformed into the coordinate system of the composite calibration object. Thus, the feature point coordinates of the accident scene were obtained. This improved the measurement accuracy when using a non-metric consumer-grade camera for traffic accident investigation. Field experiments and a specific case analysis demonstrated the feasibility of this method. The relative error using the composite calibration object for photogrammetry was far better than the results using a single calibration object, and a higher field measurement accuracy could be obtained.
